# A comprehensive characterization of metabolic signatures—hypoxia, glycolysis, and lactylation—in non-healing diabetic foot ulcers

**DOI:** 10.3389/fmolb.2025.1593390

**Published:** 2025-07-09

**Authors:** Bo Hu, Xuan Li, Yunfeng Li, Shengnan Chai, Mei Jin, Long Zhang

**Affiliations:** Wound Healing Center, Peking University Third Hospital, Beijing, China

**Keywords:** diabetic foot ulcers, metabolic reprogramming, hypoxia, glycolysis, lactylation

## Abstract

**Background and Objective:**

Diabetic foot ulcers (DFUs) are chronic complications of diabetes, driven by metabolic dysregulation and impaired wound healing. This study investigates the roles of hypoxia, glycolysis, and lactylation in DFUs and identifies potential diagnostic and therapeutic biomarkers.

**Methods:**

Single-cell RNA sequencing (scRNA-seq) was employed to assess cellular diversity, metabolic states, and intercellular communication in DFUs. KEGG/GO enrichment, pseudotime trajectory analysis, and cell-cell communication profiling were conducted to explore metabolic and cellular dynamics. Bulk RNA-seq was integrated for differential expression analysis and biomarker validation. Machine learning methods, including LASSO, Support vector machine, and Random Forest, were applied to identify and validate biomarkers across external datasets.

**Results:**

Metabolic shifts in hypoxia, glycolysis, and lactylation were observed, with keratinocytes displaying the highest metabolic activity. Pseudotime analysis revealed distinct wound-healing phases, while cell-cell communication profiling identified increased signaling among keratinocytes, fibroblasts, and SMCs in high-metabolic states, disrupting key pathways like ECM-receptor interaction and focal adhesion. Machine learning integration of scRNA-seq and bulk RNA-seq identified PKM, GAMT, and EGFR as diagnostic biomarkers strongly linked to metabolic and immune regulation. Functional analyses highlighted their roles in energy metabolism, cellular proliferation, and immune signaling, providing new insights into DFU pathogenesis.

**Conclusion:**

This study reveals metabolic dysregulation and disrupted cellular communication as central to the non-healing DFU microenvironment, with validated biomarkers and pathways offering potential targets for improved diagnosis and treatment.

## 1 Introduction

Diabetic foot ulcers (DFUs) represent a severe and complex complication of diabetes mellitus, contributing significantly to patient morbidity, reduced quality of life, and healthcare costs worldwide (Basiri et al., 2024; Mohsin et al., 2024). Despite advancements in wound care and diabetes management, DFUs remain a major clinical challenge, with chronic non-healing wounds often leading to infection, gangrene, and ultimately amputation. The multifactorial nature of DFU pathology involves a combination of neuropathy, ischemia, infection, and persistent inflammation, all of which disrupt the normal wound healing process (Manisha et al., 2024).

A central feature of DFU pathophysiology is the dysregulation of the wound microenvironment, characterized by profound metabolic disturbances, particularly hypoxia, glycolysis, and lactylation. Chronic hypoxia, arising from reduced vascular perfusion and impaired angiogenesis, is a hallmark of DFUs (Catrina and Zheng, 2016). It drives metabolic reprogramming in wound cells, shifting energy production from oxidative phosphorylation to glycolysis, a less efficient but faster pathway for ATP generation. While this shift is an adaptive response to limited oxygen availability, it also exacerbates local tissue acidosis, increases lactate production, and disrupts cellular homeostasis, thereby perpetuating the chronic wound state (Zhang et al., 2019).

Lactylation, a post-translational modification of histones mediated by lactate, has emerged as a key player in regulating macrophage polarization, gene expression, and cellular responses to metabolic stress (Zhang et al., 2019; Chen et al., 2021). Inflammatory macrophages, commonly observed in DFUs, shift toward glycolysis and lactate production, reinforcing the pro-inflammatory microenvironment and impeding the transition to a reparative M2 phenotype (Nonnenmacher and Hiller, 2018; Zhu et al., 2023). Similarly, T cells and neutrophils within DFUs demonstrate metabolic reprogramming that perpetuates inflammation, further disrupting immune homeostasis and wound resolution (Clayton et al., 2024). Beyond immune cells, fibroblasts under chronic metabolic stress exhibit excessive glycolysis and lactylation, driving aberrant extracellular matrix production and fibrosis (Henderson and O’Reilly, 2021). Keratinocytes, essential for re-epithelialization, face impaired migration and proliferation due to metabolic stress induced by hypoxia, glycolysis, and lactylation (Haller et al., 2021). These cellular dysfunctions collectively hinder angiogenesis, extracellular matrix remodeling, and tissue repair, establishing a self-perpetuating cycle of chronic inflammation and delayed wound healing in DFUs.

Recent advances in single-cell RNA sequencing (scRNA-seq) have provided unprecedented insights into the cellular and molecular heterogeneity of DFUs (Xiang et al., 2024). This high-resolution approach enables the identification of distinct cell populations, metabolic states, and intercellular communication networks, offering a deeper understanding of the dynamic processes underlying wound pathology. Metabolic states, such as hypoxia, glycolysis, and lactylation, can now be spatially and temporally mapped across specific cell types, providing critical insights into their roles in wound progression.

In this study, we utilized scRNA-seq to investigate the metabolic and cellular landscape of DFUs, with a particular focus on hypoxia, glycolysis, and lactylation. By stratifying cells into high- and low-metabolic states, we elucidated the profound impact of these metabolic pathways on cellular behavior, intercellular communication, and wound healing dynamics. Subsequent analyses in bulk RNA-seq datasets identified key diagnostic genes that may serve as biomarkers for DFU pathophysiology, offering valuable insights into the interplay between metabolism and chronic wound progression. Our findings provide a comprehensive framework for understanding the metabolic underpinnings of DFUs and highlight potential avenues for improving diagnostic and therapeutic strategies for these challenging chronic wounds.

## 2 Materials and methods

### 2.1 Data acquisition and scRNA-seq processing and analysis

Expression matrices of metabolic signature-related genes, along with relevant lesion status, were obtained from the Gene Expression Omnibus (GEO) database (https://www.ncbi.nlm.nih.gov/geo/). Specifically, GSE199939 was used as the training cohort, while GSE7014 and GSE134431 served as external validation datasets. Probe-level expression data were annotated and converted to official gene symbols using custom Perl scripts based on the platform’s annotation file. In cases where multiple probes mapped to the same gene, the median expression value was retained. Fragments per kilobase million (FPKM) values were transformed to transcripts per kilobase (TPM), which were suggested to be the same as those from microarray, according to previous description (Pachter, 2011). All expression values were subsequently log2-transformed (log2 [TPM + 1]) to stabilize variance and approximate a normal distribution.

scRNA-seq analysis was conducted in R using raw count matrices from the GSE165816 dataset. To ensure biological comparability, we included only foot skin samples from healthy non-diabetic individuals and non-healing DFU patients, excluding forearm skin and PBMC samples present in the original dataset. Raw counts were imported and processed using the Seurat package (4.4.0). Quality control filtered out cells with fewer than 200 or more than 6,000 detected genes or with >10% mitochondrial gene content. The filtered gene-cell matrix was then log-normalized, scaled, and subjected to PCA-based dimensionality reduction and UMAP visualization. Cell clustering was performed using the Louvain algorithm (resolution = 0.5), identifying 24 clusters. Cell types were annotated based on canonical marker genes provided in the original GSE165816 study (Theocharidis et al., 2022). A summary of cell type annotations and representative markers is provided in Supplementary Table S1. To further characterize macrophage subtypes, we subsetted macrophages from the integrated dataset and reprocessed them via normalization, scaling, and PCA. UMAP visualization and Louvain clustering (resolution = 0.3) were performed. M1-macrophages were annotated based on expression of CD86, CXCL9, CXCL10, and TNF; M2-like macrophages were identified via CD163 and MRC1. These markers were visualized using UMAP and dot plots to guide subtype assignment.

Proportional differences in cell-type composition between Control (healthy) and Treat (non-healing) groups were visualized using ggplot2 bar plots. DEGs in each cluster were identified using the Wilcoxon rank-sum test, with heatmaps generated via ComplexHeatmap to highlight transcriptional heterogeneity across conditions.

Metabolic states, including hypoxia, glycolysis, and lactylation, were analyzed using AUCell (1.28.0) to calculate single-cell enrichment scores. Hypoxia and glycolysis gene sets were sourced from GSEA Hallmark datasets, while lactylation-associated genes were curated from prior literature (Cheng et al., 2023). Spearman correlation coefficients were computed to quantify the associations between hypoxia, lactylation, and glycolysis and a panel of signaling pathways implicated in wound repair and immune regulation. To facilitate a qualitative visual summary, absolute correlation values were categorized into three tiers: 0–0.3 was denoted by a single plus sign (+), 0.31–0.6 by two plus signs (++), and 0.61–1.0 by three plus signs (+++). Statistical significance was conveyed via color coding: black plus signs indicated correlations with p < 0.05, while dark grey plus signs denoted non-significant associations (p ≥ 0.05). Violin plots illustrated metabolic state differences, with statistical annotations highlighting significant findings.

### 2.2 Metabolic state profiling and functional analysis

Data processing for the Treat group followed the aforementioned workflow. After quality control, normalization, and clustering as described, After performing quality control, normalization, and clustering, UMAP visualization was applied to delineate distinct clustering patterns among the identified cellular populations. Subsequent KEGG pathway enrichment analysis was performed for each identified cell type using the clusterProfiler package (4.14.6). Additionally, cell-type-specific comparisons of AUCell scores were conducted to evaluate differences in metabolic states between cells in high and low activity groups. Bar plots and violin plots were generated to visualize the proportion of cells exhibiting high or low metabolic states and to highlight statistically significant differences across cell types.

Pseudotime trajectory analysis was performed using Monocle3 (1.3.7). To define the root of the trajectory in a biologically meaningful manner, we first identified basal keratinocytes based on their high expression of canonical markers KRT14 and KRT5, which are well-established indicators of undifferentiated epidermal progenitor cells. Cells with high KRT14/KRT5 expression were selected and manually designated as the trajectory origin using the order_cells() function. Generalized Additive Models (GAM) were fitted using the mgcv package (1.9.1) to capture trends in hypoxia, glycolysis, and lactylation scores over pseudotime. Cell-type-specific metabolic trends over pseudotime were visualized using ggplot2 (3.5.1). To investigate branch-specific heterogeneity, we constructed pseudotime trajectories using Monocle3 and partitioned the resulting principal graph into three major branches based on key bifurcation points identified via Monocle3’s branch_nodes() and choose_graph_segments() functions. The number of cells and median pseudotime values within each branch were compared using ggplot2 and statistical tests. Gene Ontology (GO) enrichment analysis was performed for each pseudotime branch using branch-specific marker genes, and heatmaps of the top 10 differentially expressed genes per branch were generated to visualize molecular distinctions. Violin plots were employed to visualize differences in metabolic states among branches.

Afterwards, cells were categorized into the High-state group if all three states exhibited enrichment scores above the median, and into the Low-state group if all three states had enrichment scores below the median. The use of median thresholds to define high *versus* low states is a common and robust approach in single-cell analysis, offering simplicity and interpretability when evaluating metabolic activation patterns. Intercellular communication between high- and low hypoxia, glycolysis, and lactylation states was analyzed using CellChat (2.1.2) to quantify interaction numbers and strengths based on known ligand-receptor pairings. Communication networks were visualized, and interaction weights were compared using netVisual_circle and ggplot2. Differential signaling pathway activity was assessed using netAnalysis_signaling Role. Ligand-receptor pair analysis revealed significant differences in communication weights across states.

### 2.3 Integrative analysis for diagnostic gene identification in DFUs

Subsequently, single-cell data from DFU samples were stratified into a High-state group and an Other-state group based on hypoxia, glycolysis, and lactylation states. Cells with at least one state scoring below the median were categorized into the Other-state group. This more inclusive grouping strategy was designed to increase the number of differentially expressed genes for downstream analyses. Enrichment of inflammation-associated pathways was quantified alongside stress- and cell death–related programs using AUCell, based on curated gene sets from MSigDB (Subramanian et al., 2005) and previously published literature (Pan et al., 2024). To identify candidate transcription factors (TFs) regulating metabolic-inflammatory programs, we applied the SCENIC (1.3.1) workflow. Gene regulatory networks were inferred using GENIE3 based on the filtered expression matrix. AUCell was then used to compute regulon activity scores (AUC) for each cell. Differential regulon activity between High-state and Other-state cells was assessed using the limma package (3.62.2). To avoid redundancy and simplify interpretation, we prioritized core regulons over their extended versions when both showed statistical significance. Extended regulons were retained only when uniquely significant. The top 10 differentially active regulons (ranked by adjusted P value) were selected for downstream visualization and interpretation. Spearman correlations between AUCell scores of the top 10 differentially active TF regulons and three key metabolic programs were calculated at the single-cell level.

Differential gene expression (DEG) analysis between the High-state and Other-state groups was performed using the Wilcoxon rank-sum test. Genes with an absolute log_2_ fold change (FC) > 0.5 and p-value <0.05 were considered significantly dysregulated. In parallel, bulk RNA-seq data from the GSE199939 dataset were analyzed using the same test, followed by Benjamini–Hochberg correction to control the false discovery rate (FDR < 0.05). The intersection of DEGs from both analyses, performed using the VennDiagram package (1.7.3), served to integrate single-cell and bulk transcriptomic findings, thereby refining the identification of candidate diagnostic biomarkers.

To ensure robustness, three machine learning algorithms were applied to the GSE199939 dataset. LASSO regression was conducted using the glmnet package (4.1.8), with 10-fold cross-validation optimizing model parameters and penalization strength. A predicted probability threshold of 0.5 was used to assign class labels, consistent with standard practice in logistic regression. Random forest (RF) analysis, implemented via the randomForest package (4.7.1.2), identified genes with the highest mean decrease in classification accuracy, indicating their contribution to model performance. A classification threshold of 0.5 (i.e., ≥50% of trees voting for a class) was used to assign binary group labels. Support vector machine (SVM) modeling assigned predictive weights to genes based on a linear kernel. Class labels were derived using the default SVM decision rule, with a cutoff score of 0: samples with positive decision values were classified as DFU, and negative values as Control. The overlap of diagnostic genes identified by LASSO, random forest, and SVM was visualized in a Venn diagram to highlight shared markers.

### 2.4 Validation of diagnostic biomarkers using independent datasets

The validation of diagnostic biomarkers was conducted using three independent GEO datasets: GSE199939 (Jiang et al., 2024), GSE7014 (Vihola et al., 2010), and GSE134431 (Sawaya et al., 2020). To evaluate the diagnostic performance of the selected genes, we built a multivariate logistic regression model based on their expression profiles. Receiver Operating Characteristic (ROC) analysis was performed using the pROC package (v1.18.5) to evaluate the diagnostic performance of the combined model, with 95% confidence intervals (CIs) for AUCs computed via bootstrap resampling (2,000 iterations) using the ci.auc() function to assess the robustness and reliability of model discrimination. The diagnostic score used for ROC and AUC calculations corresponded to the predicted probability values derived from a multivariate logistic regression model constructed using the selected genes as predictors. Nomograms were constructed utilizing the rms package to illustrate the relative contributions of individual diagnostic genes to the total risk score. Additive risk scores were generated for each sample to assess the cumulative diagnostic probability. Decision curve analysis (DCA) was conducted using the rmda package (1.6) to assess the clinical utility of the combined model by calculating net benefit across a range of risk thresholds. Clinical impact curves were generated to estimate the number of high-risk predictions and their alignment with actual clinical outcomes.

### 2.5 Analysis of functional profiles, gene expression, and immune landscapes

Thereafter, the datasets GSE199939, GSE7014, and GSE134431 were integrated following standard preprocessing protocols, including batch correction using the ComBat function from the sva package (3.54.0). Weighted gene co-expression network analysis (WGCNA) was employed to construct co-expression modules. GO and Kyoto Encyclopedia of Genes and Genomes (KEGG) analyses were performed to uncover enriched biological processes and pathways. ClusterProfiler internally applies a hypergeometric test for enrichment and corrects for multiple testing using the Benjamini–Hochberg method, with adjusted p-values <0.05 considered statistically significant.

Differential expression analysis between control and treatment samples in the bulk RNA-seq dataset was performed using the Wilcoxon rank-sum test, with multiple testing correction applied via the Benjamini–Hochberg method. xCell was applied for immune infiltration profiling to estimate the relative abundance of 64 immune and stromal cell types. The analysis was performed using the xCell package (1.1.0), which applies gene signature-based enrichment and spillover compensation to deconvolute bulk transcriptomic profiles into immune cell-type–specific signals. Prior to xCell analysis, raw count data were normalized to TPM values and subsequently log2 (TPM + 1)–transformed to ensure positivity and approximate normality, as recommended for optimal algorithm performance. The correlation analysis between PKM, GAMT, EGFR, and immune cell types was performed using Spearman’s rank correlation, with heatmaps visualized using the ComplexHeatmap package (2.22.0).

### 2.6 Analysis of diagnostic gene dynamics and associations

The expression trajectories of PKM, GAMT, and EGFR along pseudotime were analyzed using the Monocle3 package to capture their dynamic changes across cellular states. Differential expression across pseudotime branches was assessed using GAMs within the mgcv package, allowing for precise modeling of gene expression trends. The Seurat package facilitated comparisons of gene expression between control and non-healing groups, while correlations with glycolysis, hypoxia, and lactylation states were quantified using AUCell to derive enrichment scores. To investigate potential functional relationships among EGFR, PKM, and GAMT, we performed protein–protein interaction analysis using the GeneMANIA platform (https://genemania.org/). The analysis was conducted in *Homo sapiens* with default settings, integrating data sources such as physical interactions, co-expression, pathway, and genetic interactions.

### 2.7 Statistical analysis

All statistical analyses were conducted in R (v4.4.2) to ensure robust and reproducible findings. Non-parametric tests were used throughout, including the Wilcoxon rank-sum test for AUCell-derived metabolic score comparisons and differential expression analysis, and Fisher’s exact test for evaluating cell-type proportion differences due to small sample sizes in some categories. For pseudotime trajectory analysis, gene expression trends across branches were assessed using the Kruskal–Wallis test, followed by Bonferroni-adjusted pairwise Wilcoxon tests. Correlations between metabolic states and gene expression were inferred through AUCell-based enrichment scoring. Statistical significance was defined as two-sided p < 0.05, and statistical significance was defined as an FDR-adjusted *p* < 0.05 using the Benjamini–Hochberg method for multiple testing correction. Multiple testing corrections were applied within each individual analysis as appropriate.

## 3 Results

### 3.1 scRNA-seq data QC and normalization

The workflow of this study is illustrated in Supplementary Figure S1. The present work acquired a total of 25,184 cells and 25,981 genes from GSE165816-derived DFU and heathy samples, which passed QC. Among these, the healthy tissue, referred to as the Control group, consisted of 18 samples and 19,367 genes, whereas the non-healing ulcer tissue, referred to as the Treat group, exhibited five samples and 9,914 genes. A standardized workflow, encompassing quality control to filter low-quality cells, normalization to account for sequencing depth, identification of highly variable genes to capture key features, scaling to standardize expression values, dimensionality reduction through principal component analysis (PCA), batch effect correction using Harmony, unsupervised clustering for group identification, and visualization via UMAP and t-SNE, was implemented for comprehensive data processing (Supplementary Figures S2A–S2F).

**FIGURE 1:**
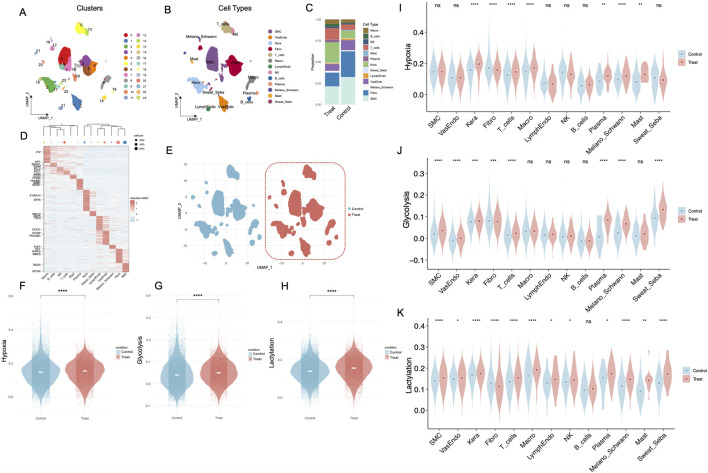
Integrated single-cell analysis reveals distinct cellular compositions, transcriptional heterogeneity, and metabolic reprogramming in DFUs between Control and Treat groups. **(A)** UMAP visualization of cell clusters derived from single-cell RNA-seq data, identifying 24 distinct cell populations from both Control and Treat groups. Each cluster is color-coded, representing transcriptionally distinct cell populations. **(B)** Cell-type annotation of the identified clusters. **(C)** Proportion of different cell types in Control and Treat groups. **(D)** Heatmap of marker gene expression across identified cell types. **(E)** UMAP embedding displaying cells stratified by condition. **(F–H)** Violin plots showing significant differences in hypoxia **(F)**, glycolysis **(G)**, and lactylation **(H)** AUCell scores between Control and Treat groups. **(I–K)** Violin plots of hypoxia **(I)**, glycolysis **(J)**, and lactylation **(K)** scores across different cell types in Control and Treat groups. DFU, diabetic foot ulcer; UMAP, uniform manifold approximation and projection. *, P < 0.05; **, P < 0.01; ***, P < 0.001; ****, P < 0.0001.

As shown in Figure 1A, 24 distinct cell clusters were identified. Subsequent cell-type annotation (Figure 1B) classified these clusters into major populations, including Smooth Muscle Cells (SMC), Vascular Endothelial Cells (VasEndo), Keratinocytes (Kera), Fibroblasts (Fibro), T Cells (T_cells), Macrophages (Macro), Lymphatic Endothelial Cells (LymphEndo), Natural Killer Cells (NK), B Cells (B_cells), Plasma Cells (Plasma), Melanocytes/Schwann Cells (Melano_Schwann), Mast Cells (Mast), Sweat and Sebaceous Gland Cells (Sweat_Seba). Further analysis (Figure 1C) revealed distinct differences in cell proportions between the Treat and Control groups, with the former showing relatively lower proportions of fibroblasts and SMCs but higher proportions of keratinocytes and T cells. Distinct marker gene expression patterns (Figure 1D) were observed across cell types, revealing clear transcriptional heterogeneity among the identified cell populations. Condition-specific UMAP plots (Figure 1E) confirmed distinct clustering of cells from the Treat and Control groups. A total of 199 hypoxia-related genes (Supplementary Table S2), 200 glycolysis-related genes (Supplementary Table S3), and 327 lactylation-related genes (Supplementary Table S4) were collected for subsequent analyses. Moreover, hypoxia (Figure 1F), glycolysis (Figure 1G), and lactylation (Figure 1H) levels were significantly elevated in the *Treat* group (**p < 0.0001**). In the hypoxia state (Figure 1I), keratinocytes, mast cells, plasma cells, melanocytes/schwann cells and macrophages display a pronounced upregulation in the Treat group compared to the Control group. Under the glycolysis condition (Figure 1J), SMCs, vascular endothelial cells, keratinocytes, T cells, plasma cells, melanocytes/schwann cells as well as sweat and sebaceous gland cells exhibit markedly elevated expression levels in the Treat group. Moreover, apart from B cells and fibroblasts, the Lactylation levels in all other cell types exhibit a consistent and substantial elevation in the Treat group relative to the Control group (Figure1K).

### 3.2 Analysis of hypoxia, glycosis and lactylation states of non-healing DFUs

**FIGURE 2:**
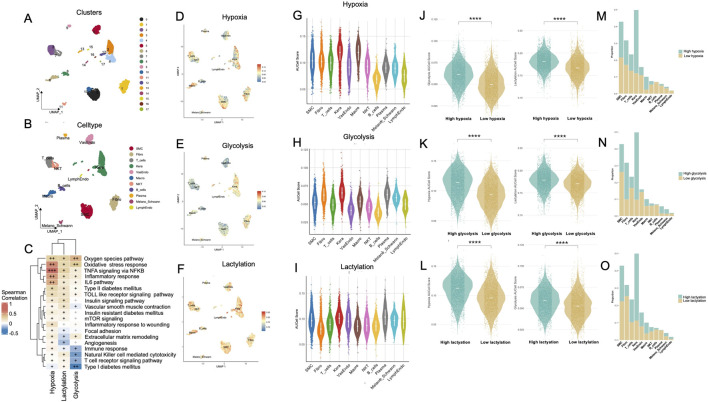
Comprehensive single-cell analysis of metabolic states reveals hypoxia, glycolysis, and lactylation differences across cell types within the Treat group of DFUs. **(A)** UMAP visualization of 17 cell clusters derived from single-cell RNA-seq data in the Treat group, with clusters color-coded to represent transcriptionally distinct populations. **(B)** Cell-type annotation of clusters. **(C)** Correlation heatmap illustrating associations between metabolic parameters (hypoxia, lactylation, glycolysis) and key signaling pathways involved in wound healing, inflammation, immune regulation, and metabolic dysregulation in non-healing diabetic foot ulcers (DFUs); correlation strength is indicated by “+” signs (|r|: 0–0.3 = +, 0.31–0.6 = ++, 0.61–1.0 = +++), with black symbols representing statistically significant correlations (p < 0.05) and dark grey representing non-significant correlations (p ≥ 0.05). **(D–F)** UMAP plots showing distribution of cells stratified by hypoxia **(D)**, glycolysis **(E)**, and lactylation **(F)** metabolic states within the Treat group. **(G–I)** Violin plots displaying AUCell scores for hypoxia **(G)**, glycolysis **(H)**, and lactylation **(I)** across different cell types. **(J–L)** Violin plots comparing hypoxia **(J)**, glycolysis **(K)**, and lactylation **(L)** scores between high and low metabolic states within the Treat group. **(M–O)** Bar charts illustrating the proportions of cells in high *versus* low metabolic states for hypoxia **(M)**, glycolysis **(N)**, and lactylation **(O)** across annotated cell types. DFU, diabetic foot ulcer; UMAP, uniform manifold approximation and projection; KEGG, Kyoto Encyclopedia of Genes and Genomes. ****, P < 0.0001.

Afterwards, UMAP visualization of the Treat group revealed distinct clustering of major cell populations—including keratinocytes, fibroblasts, smooth muscle cells (SMCs), various immune cell populations, and endothelial subsets (Figures 2A,B). Subsequent GO (Supplementary Figures S3A–S3C) and KEGG pathway enrichment analyses (Supplementary Figure S3D) further delineated diverse functional signatures and uniquely activated pathways across these cellular compartments. We also examined the correlations between three key parameters—hypoxia, lactylation, and glycolysis—and multiple signaling pathways known to influence wound repair and immune regulation. As shown in Figure 2C, hypoxia exhibited moderate to strong positive correlations with pathways regulating inflammation (e.g., TNFA signaling via NFκB, IL6 signaling, Toll-like receptor signaling) as well as those involved in extracellular matrix organization (e.g., focal adhesion, matrix remodeling) and angiogenesis. Lactylation similarly demonstrated robust positive associations, particularly with immune and cytokine-related pathways, implying that lactate-driven epigenetic modifications may contribute to the persistent inflammatory milieu in DFUs. In contrast, glycolysis exhibited mild to moderate inverse correlations with several immune-associated pathways—including the T cell receptor signaling pathway, NK cell–mediated cytotoxicity, and the broader immune response gene set—while showing positive correlations of varying magnitudes with pathways implicated in oxidative stress and those related to diabetes and insulin resistance, such as the reactive oxygen species pathway, oxidative stress response, insulin signaling pathway, and type II diabetes mellitus (Supplementary Table S5).

Cellular heterogeneity of metabolic activities across cells using AUCell scores revealed elevated hypoxia, predominantly observed in keratinocytes and macrophages (Figures 2D,G); increased glycolysis, primarily in keratinocytes and fibroblasts (Figures 2E,H); and heightened lactylation activity, notably in keratinocytes and SMCs (Figures 2F,I). Single-cell level mapping of metabolic activities across cells, based on AUCell scores, identified distinct patterns: hypoxia was predominantly elevated in keratinocytes and macrophages (Figures 2D,G); glycolysis was markedly increased in keratinocytes and fibroblasts (Figures 2E,H); and lactylation activity was notably heightened in keratinocytes and SMCs (Figures 2F,I). Among all cell types, keratinocytes consistently exhibited the highest levels across all three metabolic states, with significant differences observed compared to most other cell populations (Supplementary Figures S4A–S4C). Pairwise comparisons of metabolic states (Figures 2J–L) revealed strong interdependencies among hypoxia, glycolysis, and lactylation. Specifically, high hypoxia levels were associated with increased glycolytic activity and lactylation scores. Similarly, elevated glycolysis correlated with lactylation activity, underscoring the interplay between these processes. Lastly, Figures 2M–O compared the proportions of Control and Treat cells within high and low metabolic states.

Moreover, based on canonical marker expression, macrophage clusters were annotated as M1- or M2-subtypes. Subsequent AUCell-based metabolic scoring revealed that M1-macrophages exhibited significantly higher activity in glycolysis, lactylation, and hypoxia pathways compared to their M2-like counterparts (Supplementary Figure S5).

### 3.3 Metabolic and cellular dynamics in DFUs revealed by pseudotime analysis

**FIGURE 3:**
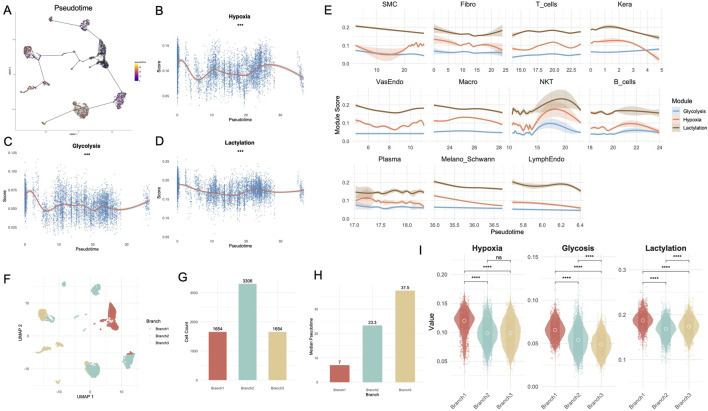
Pseudotime and branch-specific metabolic dynamics reveal distinct trajectories in the Treat group of DFUs. **(A)** Pseudotime trajectory analysis of single-cell data from the Treat group, identifying dynamic transitions between cell states. **(B–D)** GAM fits showing the trends of hypoxia **(B)**, glycolysis **(C)**, and lactylation **(D)** module scores over pseudotime. **(E)** Line plots depicting module scores for hypoxia, glycolysis, and lactylation across pseudotime in individual cell types. **(F)** UMAP visualization of cells stratified into three distinct branches (Branch 1, Branch 2, Branch 3) based on pseudotime analysis. **(G)** Bar chart showing the distribution of cell numbers across the three branches. **(H)** Median pseudotime values for each branch. **(I)** Violin plots comparing glycolysis, hypoxia, and lactylation scores across branches. DFU, diabetic foot ulcer; UMAP, uniform manifold approximation and projection; GAM, Generalized Additive Model. **, P < 0.01; ***, P < 0.001; ****, P < 0.0001.

The trajectory analysis (Figure 3A) revealed the pseudotime progression of single cells derived from DFU samples. GAM fits showed significant trends for hypoxia (Figure 3B), glycolysis (Figure 3C), and lactylation (Figure 3D). Hypoxia-related activity exhibited a biphasic trajectory: it peaked in early-stage cells, decreased during mid-pseudotime, and showed a modest resurgence in late-stage cells (p < 0.001). Glycolysis activity displayed a biphasic trend, with an initial rise during early pseudotime followed by a decline and a mild rebound in the later stage (p < 0.001). Notably, lactylation scores showed a consistently elevated profile across the trajectory, with a modest dip at mid-pseudotime followed by a recovery in the late phase. Module scores for hypoxia, glycolysis, and lactylation were analyzed across individual cell types over pseudotime (Figure 3E). Keratinocytes exhibited a distinct metabolic trajectory along pseudotime. Lactylation scores gradually declined after an early peak, while hypoxia showed a mild early elevation followed by a continuous decrease. Glycolysis remained relatively stable with a slight late-stage increase. Fibroblasts displayed stable lactylation scores across pseudotime, while hypoxia levels slightly decreased. Glycolysis remained consistently low. Macrophages showed a steady increase in hypoxia scores toward later pseudotime, whereas lactylation gradually declined. Glycolysis remained low and flat. To evaluate the activation state of wound healing programs in non-healing DFU tissue, we profiled the expression trajectories of key genes involved in epithelial repair, angiogenesis, inflammation, and matrix remodeling (e.g., KRT14, VEGFA, MMP9, COL1A1) along pseudotime (Supplementary Figure S6A). Strikingly, most healing-associated genes exhibited blunted or delayed upregulation, with relatively low and flat expression levels across pseudotime, suggesting a failure to robustly activate regenerative programs. For instance, VEGFA and PDGFB, essential for neovascularization, showed only mild expression increases.

To further dissect heterogeneity, pseudotime trajectories were divided into three branches (Branch1, Branch2, Branch3) as shown in the UMAP embedding (Figure 3F). Branch-specific cell distributions revealed significant differences, with Branch2 containing the highest number of cells (n = 3,306), followed by Branch1 (n = 1,654) and Branch3 (n = 1,654) (Figure 3G). Branch comparisons of median pseudotime values (Figure 3H) highlighted significant differences across branches, with Branch3 demonstrating the highest median pseudotime, suggesting it represents a later stage in the trajectory. Module score analysis across branches (Figure 3I) further revealed significant differences for glycolysis, hypoxia, and lactylation. All three metabolic states—hypoxia, glycolysis, and lactylation—were significantly elevated in Branch 1 compared to Branches 2 and 3 (p < 0.0001). For lactylation, levels were also significantly higher in Branch 3 than in Branch 2 (p < 0.0001), whereas glycolysis showed the opposite trend, with Branch 2 exceeding Branch 3 (p < 0.0001). However, no significant difference in hypoxia levels was observed between Branches 2 and 3. To interpret the biological relevance of the three pseudotime trajectory branches, we performed GO enrichment and analyzed the top 10 branch-specific genes (Supplementary Figure S6B). Branch 1 represented a proliferative basal-like program, enriched for epithelial structure and junctional genes such as KRT5, DSP, SFN, FXYD3, and GO terms related to epidermal development and cell adhesion (e.g., “keratinocyte differentiation”, “epidermis development”) (Supplementary Figure S7A; Supplementary Table S6). Branch 2 was characterized by fibrotic remodeling features, with top markers including COL4A1, COL6A1, THY1, SPARC. GO analysis revealed enrichment in extracellular matrix organization and tissue remodeling processes (Supplementary Figure S7B; Supplementary Table S7). Branch 3 exhibited an inflammatory immune response signature, marked by high expression of leukocyte-related genes such as PTPRC, CXCR4, CD37, and significant enrichment in GO terms like “leukocyte chemotaxis”, “T cell activation”, and “mononuclear cell proliferation” (Supplementary Figure S7C; Supplementary Table S8).

### 3.4 Differential intercellular communication and ligand-receptor interactions in hypoxia, glycolysis, and lactylation states

**FIGURE 4:**
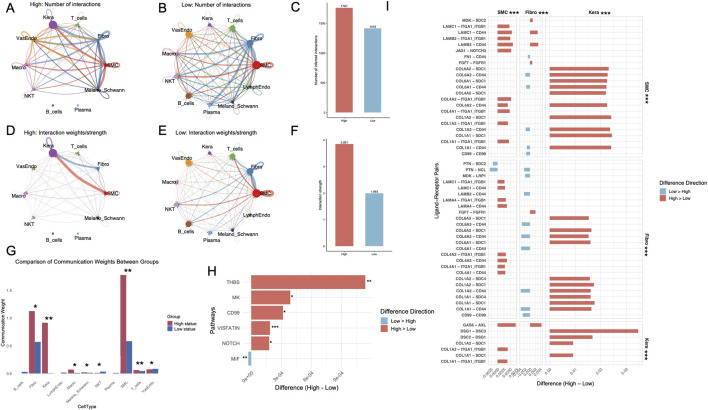
Intercellular communication analysis reveals enhanced interaction dynamics in high metabolic states of DFUs. **(A, B)** Intercellular interaction networks showing the number of interactions for high **(A)** and low **(B)** metabolic states across different cell types in DFUs. Thicker and darker edges indicate stronger interactions. **(C)** Quantification of the total number of intercellular interactions in high and low metabolic states. **(D–E)** Interaction weight networks illustrating the strength of interactions in high **(D)** and low **(E)** metabolic states. **(F)** Aggregate interaction strengths between cell types in high and low metabolic states. **(G)** Comparison of communication weights across all cell types. **(H)** Differential analysis of signaling pathways between high and low metabolic states. **(I)** Differential ligand-receptor pair expression in SMCs, fibroblasts, and keratinocytes between high and low metabolic states. DFU, diabetic foot ulcer; SMC, smooth muscle cell. *, P < 0.05; **, P < 0.01; ***, P < 0.001.

Intercellular communication was markedly altered between high and low hypoxia, glycolysis, and lactylation states. A significant increase in the number of intercellular interactions was observed in the high state compared to the low state, with specific cell types such as SMCs and keratinocytes contributing prominently to the overall interaction landscape (Figures 4A,B). Quantitative comparison further revealed that the total number of interactions in the high state was significantly greater than in the low state (Figure 4C). The strength of intercellular communication, represented by interaction weights, was consistently higher in the high state across most cell types (Figures 4D,E). This observation was further validated by aggregate interaction weights, which demonstrated a marked increase in the high state compared to the low state (Figure 4F). Interestingly, intercellular communication involving plasma cells and B cells is notably absent in the high-state group. Communication weights differed significantly between high and low states (Figure 4G). Fibroblasts, keratinocytes, and SMCs showed markedly higher weights in the high-state group (p < 0.05), while T cells, macrophages and Melano_Schwann cells exhibited minimal communication, particularly in the high-state group. Analysis of signaling pathways revealed key differences between states, with pathways such as THBS, MK, VISFATIN, and NOTCH showing statistically significant alterations (Figure 4H). In the analysis of ligand-receptor pairs, SMCs and keratinocytes displayed a predominance of interactions with higher weights in the high-state group compared to the low-state group. Conversely, fibroblasts exhibited more ligand-receptor pairs with greater communication weights in the low-state group. Notably, all pairwise comparisons of ligand-receptor interactions between cell types demonstrated statistically significant differences (p < 0.001) (Figure 4I). The comprehensive interactions between fibroblasts, keratinocytes, and SMCs, including all ligand-receptor pairings, are systematically delineated and illustrated in Supplementary Figures S8A, S8B.

### 3.5 Identification of diagnostic genes through molecular and cellular analysis in DFUs

**FIGURE 5:**
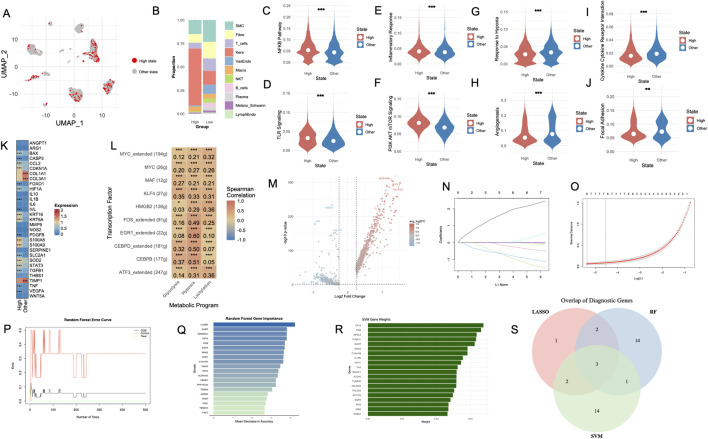
Identification of diagnostic genes through metabolic and transcriptomic analysis in DFUs. **(A)** UMAP visualization of cells stratified into high- and low-metabolic states based on hypoxia, glycolysis, and lactylation scores. **(B)** Proportions of cell types in high- and low-metabolic states. **(C–J)** Violin plots showing differential gene expression in various pathways and cellular processes between the High- and Other-states. **(K)** Heatmap displaying top differentially expressed genes between the two groups. **(L)** Spearman correlation between top transcription factor regulons and metabolic programs in High-*versus* Other-state cells. **(M)** Volcano plot illustrating differentially expressed genes between high- and low-metabolic states, with upregulated genes in red and downregulated genes in blue. **(N, O)** LASSO regression model outputs, including the coefficient path plot **(N)** and cross-validation error curve **(O)**, used for feature selection of diagnostic genes. **(P)** Random forest error curve demonstrating model accuracy for classifying control and DFU groups. **(Q)** Top-ranked genes contributing to classification accuracy in the random forest model, based on mean decrease in accuracy. **(R)** SVM model outputs highlighting gene weights, identifying key genes associated with DFUs. **(S)** Venn diagram of overlapping diagnostic genes identified through LASSO, random forest, and SVM models, pinpointing three core diagnostic biomarkers. DFU, diabetic foot ulcer; UMAP, uniform manifold approximation and projection; LASSO, least absolute shrinkage and selection operator; RF, random forest; SVM, support vector machine.

Afterwards, single-cell data from DFU samples were stratified into two groups based on levels of hypoxia, glycolysis, and lactylation (Figure 5A). The cellular composition analysis revealed notable differences in the proportion of specific cell types between the two groups, with marked shifts in the prevalence of key populations such as fibroblasts, keratinocytes, and immune cells (Figure 5B). Gene set enrichment analysis revealed significant activation of key biological pathways in the High-state group. Notably, the Nuclear Factor Erythroid 2-Related Factor 2 (NRF2) Pathway (Figure 5C), Toll-Like Receptor (TLR) Signaling (Figure 5D) and Inflammatory Response (Figure 5E) were upregulated in the High-state group. In contrast, genes involved in Extracellular Matrix (ECM) Receptor (Figure 5F), Response to Hypoxia (Figure 5G), Angiogenesis (Figure 5H), Cytokine-Cytokine Receptor Interaction (Figure 5I) and Focal Adhesion (Figure 5J) were expressed at higher levels in the Other-state group. To explore the functional implications of high metabolic states, we evaluated enrichment of eight stress- and cell death–related pathways, including oxidative stress, apoptosis, anoikis, cuproptosis, ferroptosis, autophagy, immunogenic cell death, and necroptosis. Using AUCell scoring, we found that High-state cells showed significantly higher activity across all pathways compared to Other-state cells (Supplementary Figures S9A–S9H, p < 0.001). Gene expression analysis revealed significant differences between the High- and Other-state groups (Figure 5K). Several genes, including BAX, CASP3, CCL2, CDKN1A, FOXO1 and SLC2A1, were significantly upregulated in the High-state group (p < 0.001). TIMP1, COL1A1 and COL3A1 exhibited higher expression in the Other-state group (p < 0.001). To uncover TFs that may drive the metabolic-inflammatory reprogramming of High-state cells, we applied SCENIC to reconstruct gene regulatory networks and quantify regulon activity across individual cells. Limma-based differential analysis of AUCell scores revealed a panel of transcription factors with significantly elevated activity in High-state cells compared to Other-state counterparts (Supplementary Table S9). The top 10TF regulons included MYC, KLF4, MAF, ATF3, and CEBPB—all known to orchestrate stress response, immune activation, and cellular plasticity. As shown in Figure 5L, TFs such as EGR1, FOS, CEBPB, and CEBPD exhibit consistent positive correlations with hypoxia. Notably, CEBPB and KLF4 are more strongly associated with glycolysis, while HMGB2 and ATF3 show stronger associations with lactylation.

Differential gene expression analysis between the High- and Other-state groups identified 196 significantly altered genes (Figure 5L). By intersecting these single-cell-derived genes with differentially expressed genes from the control and ulcer groups in the GSE199939 dataset, we identified 110 overlapping genes, bridging single-cell and bulk RNA-seq data to refine potential diagnostic biomarkers.

To identify robust diagnostic markers, three machine learning algorithms were employed. The LASSO regression model highlighted key genes contributing to group differentiation, as shown by the coefficient path plot and cross-validation error curve (Figures 5M,N). Random forest analysis further identified the most important genes driving classification based on mean decrease in accuracy, emphasizing the contributions of inflammatory and extracellular matrix-related genes (Figures 5O,P). Similarly, the SVM algorithm assigned significant weights to genes associated with cellular stress and metabolic pathways (Figure 5Q). The overlap of diagnostic genes across these three approaches was visualized using a Venn diagram (Figure 5R), revealing a core set of shared markers, including EGFR, GAMT, and PKM, which were identified as pivotal genes. The corresponding model coefficients and feature weights for each algorithm have been provided in Supplementary Tables S10–S12.

### 3.6 Robust validation of diagnostic biomarkers across independent cohorts

**FIGURE 6:**
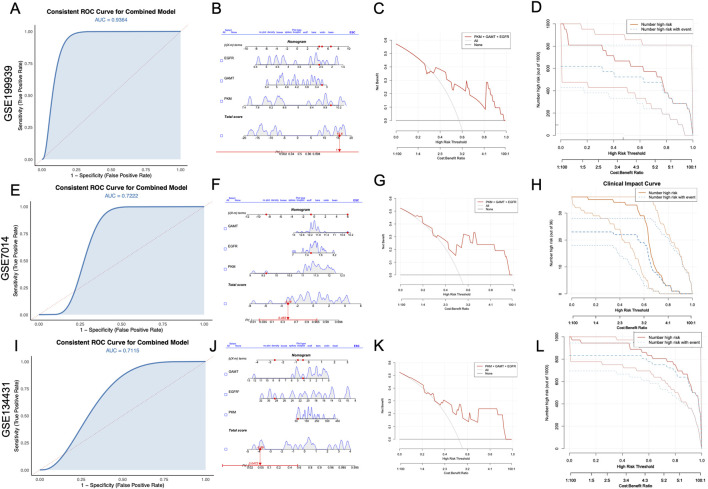
Validation of diagnostic performance of combined biomarkers (PKM, GAMT, and EGFR) across independent datasets. **(A, E, I)** ROC curves for the combined model demonstrate consistent diagnostic performance with AUC values of 0.9364 (GSE199939), 0.7222 (GSE7014), and 0.7115 (GSE134431). **(B, F, J)** Nomograms illustrating the individual contributions of PKM, GAMT, and EGFR to the total diagnostic risk score in each dataset. **(C, G, K)** Decision curve analyses showing the net clinical benefit of the combined biomarker model compared to baseline scenarios (“none” and “all”). **(D, H, L)** Clinical impact curves illustrating the relationship between high-risk predictions and actual events, validating the clinical applicability of the combined model in all three datasets. AUC, area under the curve; ROC, receiver operating characteristic; DFU, diabetic foot ulcer.

The diagnostic potential of the selected biomarkers was systematically validated using three independent GEO datasets. The ROC curves (Figures 6A,E,I) exhibited compelling predictive performance for the combined model, yielding AUC values of 0.9364, 0.7222, and 0.7115 for GSE199939, GSE7014, and GSE134431, respectively. The nomograms (Figures 6B,F,J) elucidate the contributions of individual genes to the total diagnostic risk score. The additive risk model highlights a consistent alignment of higher total scores with an increased probability of diagnostic classification, signifying the synergistic contribution of these genes within the predictive framework. Decision curve analysis (Figures 6C,G,K) provided a robust assessment of the model’s clinical utility by evaluating net benefit across a range of risk thresholds. The combined model consistently demonstrated higher net clinical benefit compared to the “treat-all” or “treat-none” strategies across a wide range of threshold probabilities, particularly between 0.3 and 0.8. In parallel, CIC results (Figures 6D,H,L) showed that the number of predicted high-risk individuals closely matched the number of actual DFU cases. Across all three datasets, the combined model demonstrated robust performance in identifying individuals at high risk, consistently showcasing its predictive accuracy and reliability across independent cohorts.

### 3.7 Comprehensive functional profiling, differential expression analysis, and immune landscape characterization of diagnostic genes in DFUs

**FIGURE 7:**
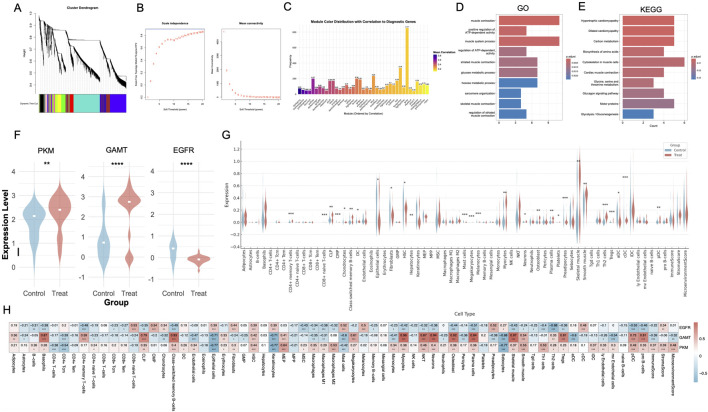
Diagnostic gene enrichment analysis, differential expression, and immune-related profiling in DFUs. **(A)** Hierarchical clustering dendrogram from WGCNA, identifying co-expression modules associated with DFUs. **(B)** Scale-free topology model fit and mean connectivity plot determining the optimal soft-thresholding power for network construction. **(C)** Bar plot showing the correlation of co-expression modules with diagnostic genes, highlighting the darkgrey module as the most significantly correlated. **(D, E)** GO and KEGG pathway enrichment analysis of the yellow module, revealing significant associations with metabolic and muscle-related pathways critical to wound healing. **(F)** Violin plots showing the differential expression of PKM, GAMT, and EGFR between Control and Treat groups, with upregulation of PKM and GAMT and downregulation of EGFR in DFUs. **(G)** Violin plots displaying cell-type-specific expression levels of diagnostic genes, highlighting keratinocytes, fibroblasts, and immune cells. **(H)** Heatmap of correlations between diagnostic genes (PKM, GAMT, EGFR) and immune cell types, showing distinct patterns of association with wound healing and inflammation-related cells. WGCNA, weighted gene co-expression network analysis; GO, Gene Ontology; KEGG, Kyoto Encyclopedia of Genes and Genomes. *, P < 0.05; **, P < 0.01; ***, P < 0.001; ****, P < 0.0001.

To further validate the diagnostic significance of PKM, GAMT, and EGFR, we performed an integrated analysis of datasets GSE199939, GSE7014, and GSE134431. WGCNA identified several co-expression modules associated with the diagnostic genes. Among these, the darkgrey module stood out with the strongest correlation to PKM, GAMT, and EGFR (correlation coefficient of 0.83; Figures 7A–C). Given its robust association, the darkgrey module was prioritized for further investigation. Functional enrichment analysis of the darkgrey module revealed its involvement in processes highly relevant to DFU pathology. GO analysis identified key processes such as sarcomere organization, glucose metabolism, and muscle contraction (Figure 7D). KEGG pathway enrichment further highlighted dysregulation in glycolysis/gluconeogenesis and cardiac muscle contraction pathways (Figure 7E). In the integrated dataset, differential expression analysis revealed that PKM and GAMT were significantly upregulated in DFU lesions, while EGFR was predominantly expressed in control tissues (Figure 7F).

Additionally, xCell-based immune infiltration profiling demonstrated notable differences in the immune landscape between DFU and control tissues. Specifically, DFU tissues exhibited increased populations of Tregs, Th2 cells, Tgd cells, and CD4^+^ memory T cells, alongside reduced levels of fibroblasts, epithelial cells, and HSCs (Figure 7G). Correlation analysis between PKM, GAMT, EGFR, and immune cell types revealed that PKM and GAMT were positively correlated with most immune cells, while EGFR exhibited negative correlations (Figure 7H).

### 3.8 Pseudotime and differential expression analysis of diagnostic genes in scRNA-seq data

**FIGURE 8:**
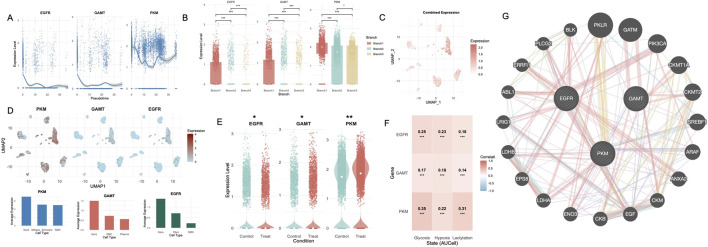
Pseudotime analysis, differential expression, and metabolic correlations of diagnostic genes in single-cell data. **(A)** Pseudotime trajectories of PKM, GAMT, and EGFR expression levels, highlighting distinct temporal patterns associated with metabolic states. **(B)** Boxplots illustrating significant expression differences for PKM, GAMT, and EGFR across pseudotime-defined branches. **(C)** Combined expression levels of diagnostic genes mapped onto the UMAP. **(D)** UMAP visualization showing cell-specific expression patterns of PKM, GAMT, and EGFR within the Treat group. Bar plots displaying the top three cell types with the highest average expression for each gene. **(E)** Violin plots of PKM, GAMT, and EGFR, displaying significant differential expression between Control and Treat groups in single-cell data. **(F)** Heatmap of correlations between diagnostic gene expression and metabolic states (glycolysis, hypoxia, and lactylation). **(G)** PPI network of metabolism-related genes enriched in non-healing DFU tissues. UMAP, uniform manifold approximation and projection; DFU, diabetic foot ulcer; PPI,Protein-protein interaction **, P < 0.01; ***, P < 0.001.

In the scRNA-seq dataset GSE165826, the expression trajectories of PKM, GAMT, and EGFR were analyzed along pseudotime, revealing distinct temporal patterns (Figure 8A). EGFR expression exhibited a high initial level, rapidly declined in early pseudotime, and remained suppressed throughout the trajectory with only minor fluctuations. GAMT exhibited a sharp decline in expression after an initial high level in early pseudotime. While its expression remained low throughout most of the trajectory, a subtle upturn was observed at the terminal stage. PKM, in contrast, showed a sustained and dynamic profile. After an initial decline, expression rebounded in mid-pseudotime and gradually increased again at late pseudotime. Stratified by pseudotime branches, all three genes—PKM, GAMT, and EGFR—exhibited the highest expression levels in Branch 1 (Figure 8B). All three genes exhibited significant differential expression across pseudotime branches.

Regional mapping of gene expression in the non-healing subgroup of the single-cell dataset revealed differential distribution patterns of PKM, GAMT, and EGFR across cell clusters (Figures 8C,D). PKM expression was highest in keratinocytes, followed by melanocyte/Schwann cells and SMCs. GAMT expression was also predominantly observed in keratinocytes, with lower levels in SMCs and plasma cells. EGFR expression was most prominent in keratinocytes, with moderate expression in fibroblasts and SMCs. When comparing the control and non-healing groups, PKM and GAMT were significantly upregulated in the non-healing group, while EGFR exhibited a contrasting downregulation (Figure 8E). Correlation analysis between the diagnostic genes and glycolysis, hypoxia, and lactylation states in the non-healing group revealed strong and statistically significant associations. PKM displayed the highest correlations across all states, particularly with hypoxia (R = 0.35, p < 0.001). EGFR showed moderate correlations with these states, while GAMT demonstrated weaker yet significant correlations (Figure 8F). To assess the potential synergy and complementarity among the three selected genes, we constructed a functional network using GeneMANIA. The resulting network showed that EGFR is embedded in a signaling hub connected to downstream effectors such as PIK3CA, ABL1, and EPS8, highlighting its role in epithelial proliferation and signal transduction. PKM clustered with glycolytic enzymes including LDHA, ENO3, and PKLR, indicating its metabolic function in glucose processing and lactate production. In contrast, GAMT formed a discrete module with mitochondrial creatine kinases (e.g., CKMT1A, CKMT2), involved in cellular energy buffering. While GAMT lacked direct interaction with the other two genes, PKM and EGFR were connected through multiple intermediary nodes (Figure 8G).

## 4 Discussion

DFUs represent a severe and debilitating complication of diabetes mellitus, characterized by chronic, non-healing wounds that result from a complex interplay of metabolic, vascular, and immunological dysregulation (Zhang et al., 2024a). Despite advances in wound care, DFUs remain a leading cause of lower extremity amputations, necessitating a deeper understanding of their molecular underpinnings to identify effective diagnostic and therapeutic targets. In this study, leveraging both single-cell and bulk transcriptomic datasets, we identified key diagnostic biomarkers—PKM, GAMT, and EGFR—and elucidated their roles in metabolic and immune remodeling within the DFU microenvironment.

Initially, scRNA-seq analysis comprehensively provided a high-resolution perspective on the metabolic heterogeneity within DFU lesions. Hypoxia, glycolysis and lactylation are deeply intertwined in the pathophysiology of non-healing DFUs, reflecting critical disruptions in the metabolic and immune microenvironments. Hypoxia, driven by impaired vascularization, stabilizes hypoxia-inducible factor 1-alpha (HIF-1α), which enhances glycolytic flux through enzymes such as PFKFB3, allowing cells to adapt to oxygen deprivation by prioritizing glycolytic ATP production (Irizarry-Caro et al., 2020; Zhang et al., 2021a). However, the adaptation becomes pathological in DFUs, with excessive glycolysis fueling lactate accumulation, chronic inflammation, and insufficient angiogenesis (Dichtl et al., 2021; Yang et al., 2017). Lactylation, as both a metabolic and epigenetic response to glycolysis, further complicates wound healing in DFUs. Elevated lactate levels not only stabilize HIF-1α and induce VEGF signaling but also modify histones, influencing macrophage polarization and tissue repair gene expression (Dichtl et al., 2021). While lactylation promotes reparative M2 macrophage phenotypes under physiological conditions, its dysregulation in DFUs perpetuates inflammatory imbalance and delays resolution (Alvarez et al., 2021). This maladaptive metabolic-immune feedback loop sustains a hypoxic, inflammatory microenvironment, preventing effective angiogenesis and tissue regeneration. Our comprehensive correlation analyses further reveal that the upregulation of hypoxia, lactylation, and glycolysis is intricately linked to multiple signaling pathways that govern wound repair and immune regulation in non-healing DFUs. The robust positive correlations between hypoxia and pathways involved in inflammation, extracellular matrix organization, and angiogenesis suggest that chronic hypoxia may trigger compensatory repair responses, yet simultaneously perpetuate maladaptive proinflammatory cascades, thereby impeding effective wound resolution. Similarly, the strong positive associations between lactylation and cytokine-related and immune pathways are in line with reports that H3K18 lactylation activates NF-κB signaling and induces proinflammatory cytokines such as IL-6 and IL-8 (Wei et al., 2023a). This supports the hypothesis that lactylation may promote a sustained proinflammatory state by skewing macrophages toward an M1-like phenotype in DFUs (Zhou et al., 2022). Furthermore, glycolysis exhibited mild to moderate inverse correlations with several immune-associated pathways, suggesting that heightened glycolytic flux may concomitantly dampen effective immunoregulatory mechanisms (Zhu et al., 2025). Concurrently, positive correlations were observed between glycolysis and pathways related to oxidative stress, hyperglycemia, and insulin resistance, implying that increased glycolytic activity not only reflects but may further exacerbate the pro-oxidative, hyperglycemic, and insulin-resistant conditions characteristic of DFUs.

Moreover, keratinocytes demonstrated the highest levels of hypoxia, glycolysis, and lactylation in non-healing DFU lesions, likely due to their pivotal role in wound healing and re-epithelialization. Keratinocytes are metabolically active, requiring substantial energy for migration, proliferation, and extracellular matrix remodeling under ischemic and nutrient-deprived conditions (Zhang et al., 2024b; Piipponen et al., 2020). Hypoxia drives keratinocytes to upregulate glycolysis as a compensatory mechanism, while lactylation may regulate gene expression critical for tissue repair. However, dysregulation of this heightened metabolic activity may exacerbate chronic wound pathology, disrupting the delicate balance required for proper tissue repair and contributing to sustained inflammation and progressive tissue damage. Similarly, the elevated glycolytic, hypoxic, and lactylation activities observed in M1-like macrophages are indicative of their pro-inflammatory and metabolically active phenotype. In the context of non-healing DFUs, such metabolic reprogramming may signify chronic inflammatory activation and insufficient adaptation to the hostile wound microenvironment (Xu et al., 2024). Sustained hypoxia and metabolic stress can stabilize HIF-1α, reinforcing glycolytic flux and driving M1 polarization, thereby creating a self-amplifying cycle of inflammation and tissue damage that impedes wound resolution (Wu et al., 2023). Collectively, the interplay of hypoxia, glycolysis, and lactylation establishes a self-perpetuating cycle of metabolic dysfunction and immune dysregulation in DFUs.

Pseudotime analysis of non-healing DFUs revealed distinct metabolic dynamics across key cell types. In macrophages, glycolysis levels are elevated during early pseudotime, reflecting M1 activation via the HIF-1α/PKM2 axis, which promotes pro-inflammatory responses through glycolytic reprogramming and IL-1β production (Corcoran and O’Neill, 2016; Palsson-McDermott et al., 2015). Persistently low lactylation levels, however, indicate an impaired M1-to-M2 transition, critical for resolving inflammation (Forteza et al., 2023; Semba et al., 2016). This disrupted metabolic-epigenetic axis, characterized by inadequate macrophage polarization, exacerbates chronic inflammation and delays wound healing in DFUs (Iniesta et al., 2001). Keratinocytes exhibited sustained lactylation early in pseudotime, aligning with their role in promoting angiogenesis via lactylation-mediated stabilization of HIF-1α (Kato et al., 2021). However, the muted hypoxia and glycolysis levels suggest impaired metabolic flexibility in the hypoxic DFU microenvironment, potentially limiting epithelial migration and tissue closure. Fibroblasts demonstrated a late-stage surge in the three metabolic states, indicative of metabolic adaptation to extracellular matrix production. Yet, relatively low hypoxia levels throughout pseudotime may suggest inadequate activation of angiogenic or granulation functions (Li et al., 2021). The differential glycolysis, hypoxia, and lactylation levels across pseudotime branches reflect distinct metabolic states and temporal dynamics in non-healing DFUs. Branch 1, with the highest metabolic activity, likely represents an early, hypoxia-driven adaptive response aimed at wound closure through glycolysis, lactate signaling, and inflammatory pathways such as MAPK and Rap1 signaling. In contrast, Branch 2, characterized by the lowest metabolic activity, reflects a later, metabolically quiescent or dysregulated state with impaired reparative capacity, as evidenced by pathways related to ECM-receptor interactions and focal adhesion, indicative of inadequate matrix remodeling and tissue repair. Branch 3 occupies an intermediate position, reflecting a transitional phase with partial metabolic adaptation. These findings underscore the temporal and cellular heterogeneity of metabolic responses in DFUs, highlighting the disrupted coordination of metabolic states across time as a key barrier to effective healing.

The augmented intercellular communication between fibroblasts and keratinocytes, as well as SMCs and keratinocytes, observed in high metabolic states, underscores an intensified metabolic response aimed at mitigating the severe stress within the non-healing DFU microenvironment. In SMCs, GAS6–AXL was among the most enriched pairs in High-state cells, consistent with its known role in vascular remodeling and anti-inflammatory signaling. Additionally, several extracellular matrix (ECM)-integrin pairs—LAMC1–CD44, LAMB2–CD44, COL4A2–ITGA1–ITGB1, and COL4A1–ITGA1–ITGB1—were activated, suggesting enhanced ECM interaction and potential migration readiness in metabolically active SMCs (Barbosa et al., 2024). In fibroblasts, the top L–R pairs included GAS6–AXL, along with multiple collagen–CD44 interactions (COL6A2–CD44, COL6A1–CD44, COL1A1–CD44, COL1A2–CD44). These signals are tightly linked to matrix production, fibroblast activation, and wound fibrosis, highlighting their pathogenic relevance in chronic wound states (Yang et al., 2025; Zhang et al., 2022). In keratinocytes, top-ranked ligand–receptor pairs such as COL1A1–SDC1, COL6A2–SDC1, COL1A2–SDC1 and DSG1–DSC3 were elevated in the High-state group. These interactions are essential for epithelial–mesenchymal adhesion, cell–matrix contact, and barrier integrity (Wei et al., 2023b; Huang et al., 2023). These ligand–receptor interactions have been extensively studied in tumor biology, yet their specific roles in the context of DFU remain largely unexplored and warrant further investigation. Fibroblast-keratinocyte interactions likely promote ECM synthesis and keratinocyte migration, processes essential for initiating re-epithelialization and wound closure (Russo et al., 2020). Concurrently, the elevated crosstalk between SMCs and keratinocytes may drive angiogenic pathways, potentially mediated by the secretion of hypoxia-responsive pro-angiogenic factors such as VEGF via HIF-1α activation (Liu et al., 2023). However, the chronic hypoxia and dysregulated metabolic states characteristic of DFUs may shift these adaptive interactions toward pathological outcomes (Manchanda et al., 2023). Excessive ECM deposition from hyperactive fibroblast signaling may lead to fibrosis, while aberrant SMC-driven vascular remodeling may result in dysfunctional angiogenesis and capillary leakage (Xiong and Liu, 2017). Additionally, the sustained pro-inflammatory milieu, compounded by unresolved metabolic stress, perpetuates tissue damage and hinders resolution of the wound (Alfaro et al., 2022). Thus, while the enhanced communication between these cell types reflects an inherent attempt to restore homeostasis, it paradoxically exacerbates the chronic wound pathology in DFUs under conditions of severe metabolic dysregulation.

Subsequent investigations have demonstrated that DFU cells exhibiting more pronounced hypoxic, glycolytic, and lactylation states are characterized by enhanced chronic inflammation and cellular stress. In contrast, cells from the Other-state group exhibit superior capabilities in tissue repair, adaptation to hypoxic environments, promotion of neovascularization, regulation of inflammation, and enhancement of cellular adhesion and migration. Cells in the High-state exhibited significantly elevated enrichment of multiple cell death–associated programs. This coordinated activation of diverse death pathways suggests a highly stressed or immunologically engaged microenvironment, in which parallel mechanisms converge to restrict cell survival (Li et al., 2024). These observations imply that the High-state may represent a metabolically and immunologically vulnerable cell population undergoing active elimination or transition. Gene-related analysis corroborated these findings. Genes implicated in the promotion of chronic inflammation (Schonthaler et al., 2013; Christmann et al., 2021; Zhang et al., 2021b), including S100A8, S100A9, CCL2, and IL1B, as well as those associated with oxidative stress (Gao et al., 2020; Li and Gao, 2023; Hori et al., 2013), such as SOD2 and FOXO1, were markedly upregulated in the High-state. Additionally, genes like BAX and CASP3, which are known to mediate apoptosis (Kiraz et al., 2016), were also highly expressed, potentially contributing to the apoptotic processes that may lead to keratinocyte and endothelial cell death. Furthermore, genes related to disrupted keratinization and impaired epidermal differentiation (Grzanka et al., 2012; Dull et al., 2023), such as KRT16, KRT6A, and IVL, were found to be elevated in the High-state, suggesting a pathological exacerbation of chronic wound healing. In contrast, genes involved in the inhibition of matrix metalloproteinases (MMPs) and the promotion of collagen deposition (Yim et al., 2018; Ana et al., 2025), namely TIMP1, COL1A1, and COL3A1, exhibited significantly higher expression levels in the Other-state, indicative of enhanced tissue repair and fibrosis processes. Moreover,the top enriched TFs—including MYC, KLF4, ATF3, and CEBPB—have established roles in glycolysis, oxidative stress response, and immune signaling via TLR and NFκB pathways (Bhawe and Roy, 2018; Chen et al., 2022; Nabar and Kehrl, 2017). For instance, MYC promotes glycolytic gene expression (Bian et al., 2022; Yeung et al., 2008); KLF4 and ATF3 mediate TLR feedback (Kim et al., 2010; Liang et al., 2024); and CEBPB directly regulates pro-inflammatory cytokines such as IL-6 and TNF (Ren et al., 2023; Kim et al., 2009). MAF and HMGB2 further contribute to immune tolerance and DAMP-related activation (Fan et al., 2024; Koto et al., 2022).

The integration of single-cell and bulk transcriptomic analyses established PKM, GAMT, and EGFR as robust diagnostic biomarkers, with their performance validated across multiple independent datasets. Nomograms constructed from these genes provided a clinically interpretable framework for risk stratification, while decision and clinical impact curves demonstrated the practical utility of these biomarkers in identifying high-risk cases. The robust diagnostic accuracy, reflected by high AUC values in multiple datasets, underscores the potential of these markers for clinical translation.

PKM, a critical enzyme in the glycolytic pathway, exerts profound control over cellular energy metabolism and metabolite production (Yang et al., 2012). Under hypoxic conditions, PKM activity is likely altered, driving an upregulation of glycolysis and subsequent lactate accumulation. The excess lactate not only exacerbates local acidosis, impairing cellular functionality, but also acts as a signaling molecule through lactylation modifications. These effects collectively exacerbate the pathological progression of DFUs. GAMT, a key enzyme in the creatine biosynthesis pathway, is essential for maintaining cellular energy metabolism and homeostasis (Baker et al., 2021). In DFUs, upregulated GAMT likely represents a compensatory response to heightened energy demands and metabolic stress. As a key enzyme in creatine biosynthesis, GAMT supports intracellular energy reservoirs (Mercimek-Mahmutoglu et al., 2006; Curt et al., 2015). However, the chronic hypoxic and inflammatory microenvironment may impair creatine utilization or disrupt downstream energy metabolism, limiting its reparative potential. EGFR plays a fundamental role in cellular proliferation and signal transduction (Wee and Wang, 2017). Hypoxia and metabolic dysregulation associated with DFUs may result in suppression of the EGFR signaling pathway (Mamo et al., 2020). Excessive glycolysis and lactylation may potentially alter the extracellular microenvironment or post-translational modifications, which could affect EGFR-ligand interactions. However, direct mechanistic evidence for such disruption remains to be established. Collectively, these findings highlight the distinct but complementary roles of PKM, GAMT, and EGFR in the non-healing DFU microenvironment. PKM and GAMT, while orchestrating metabolic adjustments to the hypoxic and glycolytic milieu of DFUs, paradoxically fail to facilitate wound resolution, reflecting the persistent metabolic and inflammatory dysfunction. In contrast, EGFR, with its diminished expression in lesion sites, may signify impaired tissue repair mechanisms and a disrupted equilibrium in the wound microenvironment, further perpetuating chronic pathology. The coordinated dysregulation of these genes not only mirrors the metabolic and inflammatory dysfunction underlying DFU pathophysiology, but also suggests their potential as novel, mechanistically informed biomarkers beyond classical inflammatory markers.

These findings may provide a rationale for metabolic-targeted therapies in DFUs. For instance, PKM2 inhibitors have been shown to attenuate inflammation and improve macrophage polarization (Xiang et al., 2025), while modulation of lactylation—though still in early-stage research—represents a promising avenue for reprogramming chronic inflammation. In parallel, strategies aimed at restoring EGFR signaling, such as topical EGF application, have demonstrated clinical benefit in wound repair and may warrant reconsideration in DFUs with diminished EGFR expression (Shakhakarmi et al., 2023). Although our study is descriptive in nature and lacks experimental validation, it lays a robust foundation for future mechanistic and interventional investigations into metabolic reprogramming as a therapeutic strategy for chronic wound healing.

Notably, the xCell deconvolution revealed increased enrichment of Tregs, Th2 cells, dendritic cells (DCs), and several other immune and stromal cell types in DFUs. Interestingly, while previous studies (Dawi et al., 2025) have reported reduced numbers or impaired function of Tregs in diabetic patients, our xCell-based deconvolution revealed an increased enrichment of Tregs in non-healing DFUs compared to healing tissues. This apparent discrepancy may reflect a compensatory accumulation of Tregs in response to persistent, unresolved inflammation. Despite their increased abundance, these Tregs may be functionally exhausted or unable to effectively suppress chronic inflammatory signals in the wound microenvironment. Alternatively, their expansion could contribute to an overly suppressive immune milieu, limiting the activity of effector immune cells necessary for infection control and tissue regeneration (Alvarez et al., 2020). Th2 cells, through cytokines such as IL-4 and IL-13, can promote fibrosis and skew macrophage polarization toward anti-inflammatory M2-like states (Allen and Wynn, 2011), which may contribute to persistent chronic inflammation, fibrotic tissue remodeling, and impaired re-epithelialization in DFUs. Collectively, our study uniquely integrates single-cell and bulk transcriptomic data to uncover the coordinated roles of hypoxia, glycolysis, and lactylation in DFUs—three interrelated metabolic programs that have not been jointly explored in this disease context and uncovers a previously unrecognized metabolic gene signature with potential diagnostic relevance.

While this study provides valuable insights into the molecular and cellular mechanisms underlying DFUs, several limitations should be addressed. First, the reliance on cross-sectional data limits the ability to capture temporal dynamics of wound healing and disease progression. Longitudinal studies are needed to delineate causal relationships and identify stage-specific therapeutic targets. Second, the functional roles of PKM, GAMT, and EGFR in DFU pathogenesis require further validation through *in vitro* and *in vivo* studies. Finally, while the integration of single-cell and bulk RNA-seq data enhances the robustness of our findings, additional datasets and larger sample sizes are warranted to validate these results in diverse patient populations.

## 5 Conclusion

In conclusion, this study highlights the intricate interplay between metabolic dysregulation, cellular communication, and molecular dysfunction in the pathogenesis of non-healing DFUs. Hypoxia, glycolysis, and lactylation states were found to be pivotal drivers of cellular and molecular alterations, with significant impacts on fibroblasts, keratinocytes, and smooth muscle cells, which exhibited enhanced intercellular communication in high metabolic states. PKM and GAMT highlight metabolic stress in DFUs, while EGFR downregulation signals impaired tissue repair and disrupted homeostasis. Together, these findings provide critical insights into the contrasting roles of these genes in DFU pathology and their potential as diagnostic markers and therapeutic targets.

## Data Availability

The datasets presented in this study can be found in online repositories. The names of the repository/repositories and accession number(s) can be found in the article/Supplementary Material.
