# Supplementary figures and images for: A comprehensive characterization of metabolic signatures—hypoxia, glycolysis, and lactylation—in non-healing diabetic foot ulcers
Source: Front Mol Biosci. 2025 Jul 9;12:1593390. doi: 10.3389/fmolb.2025.1593390 (PMC12284369; doi:10.3389/fmolb.2025.1593390)

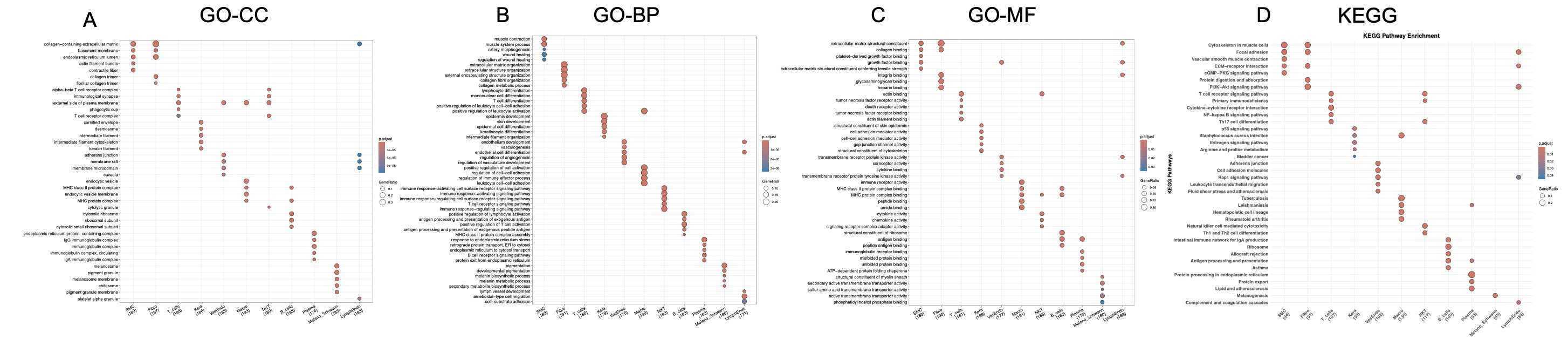

Supplement: Supplementary file 2 [file Image3.jpeg]

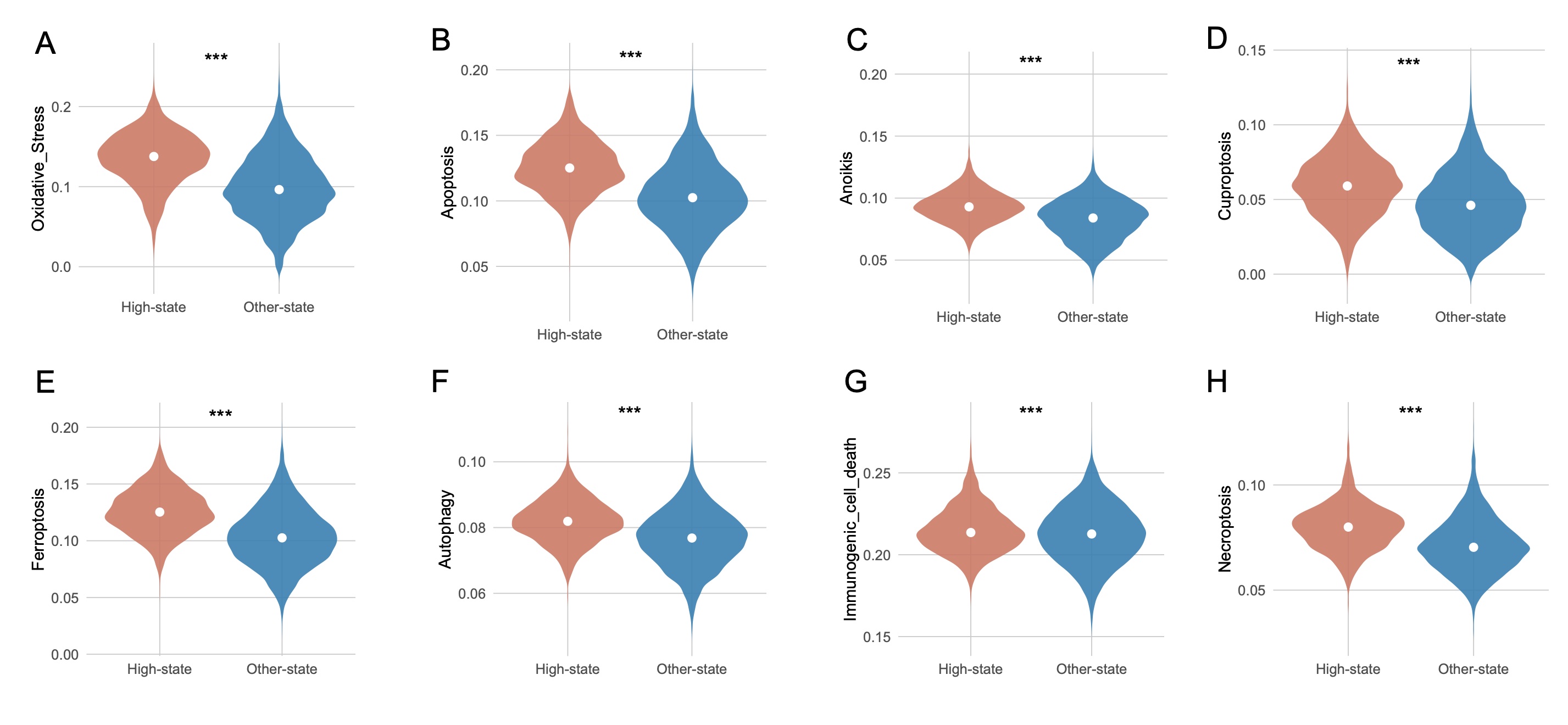

Supplement: Supplementary file 5 [file Image9.jpeg]

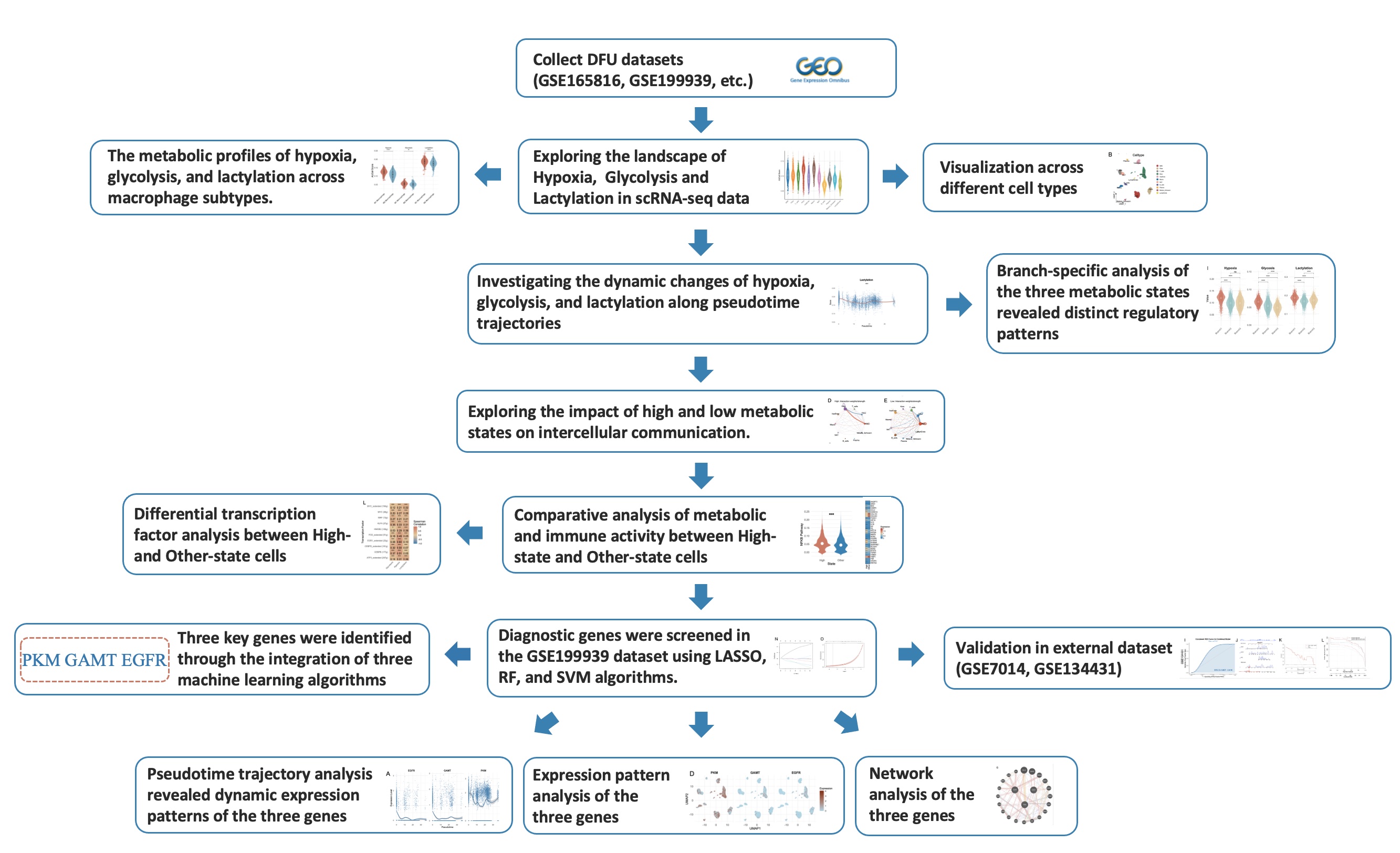

Supplement: Supplementary file 7 [file Image1.jpeg]

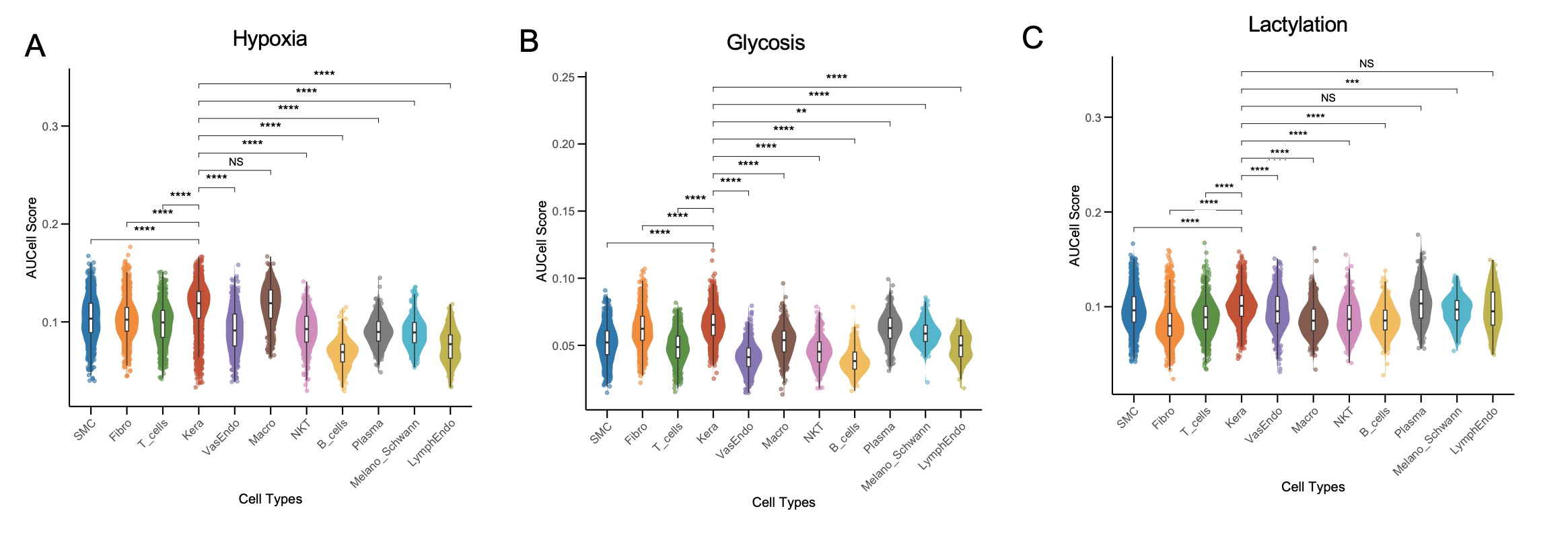

Supplement: Supplementary file 8 [file Image4.jpeg]

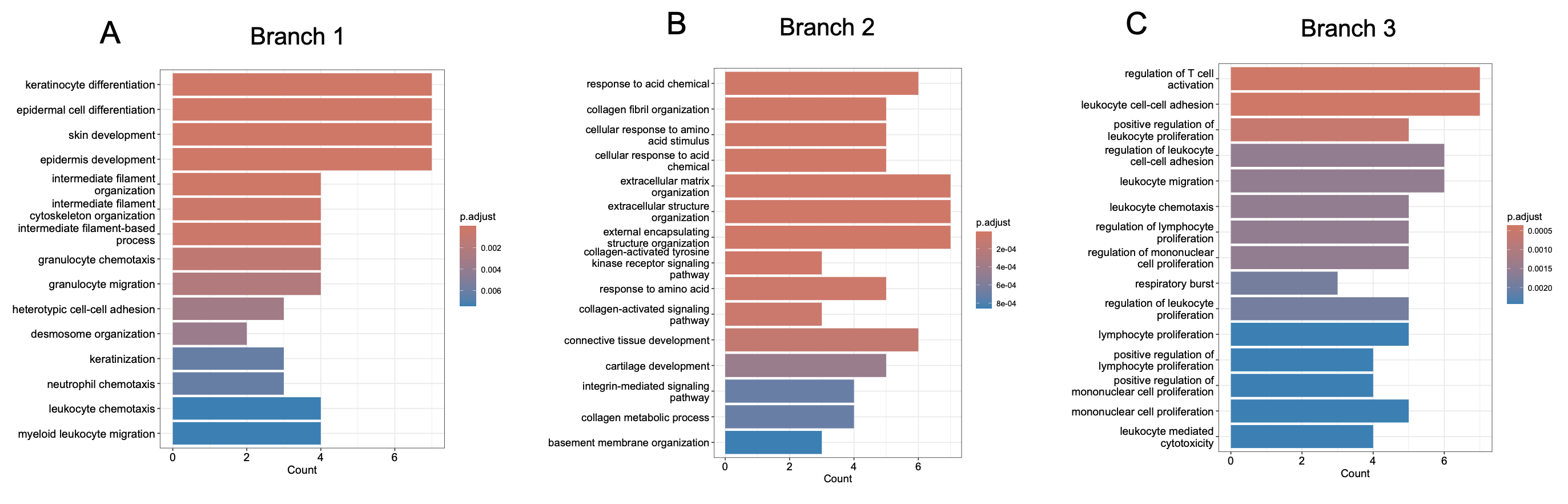

Supplement: Supplementary file 9 [file Image7.jpeg]

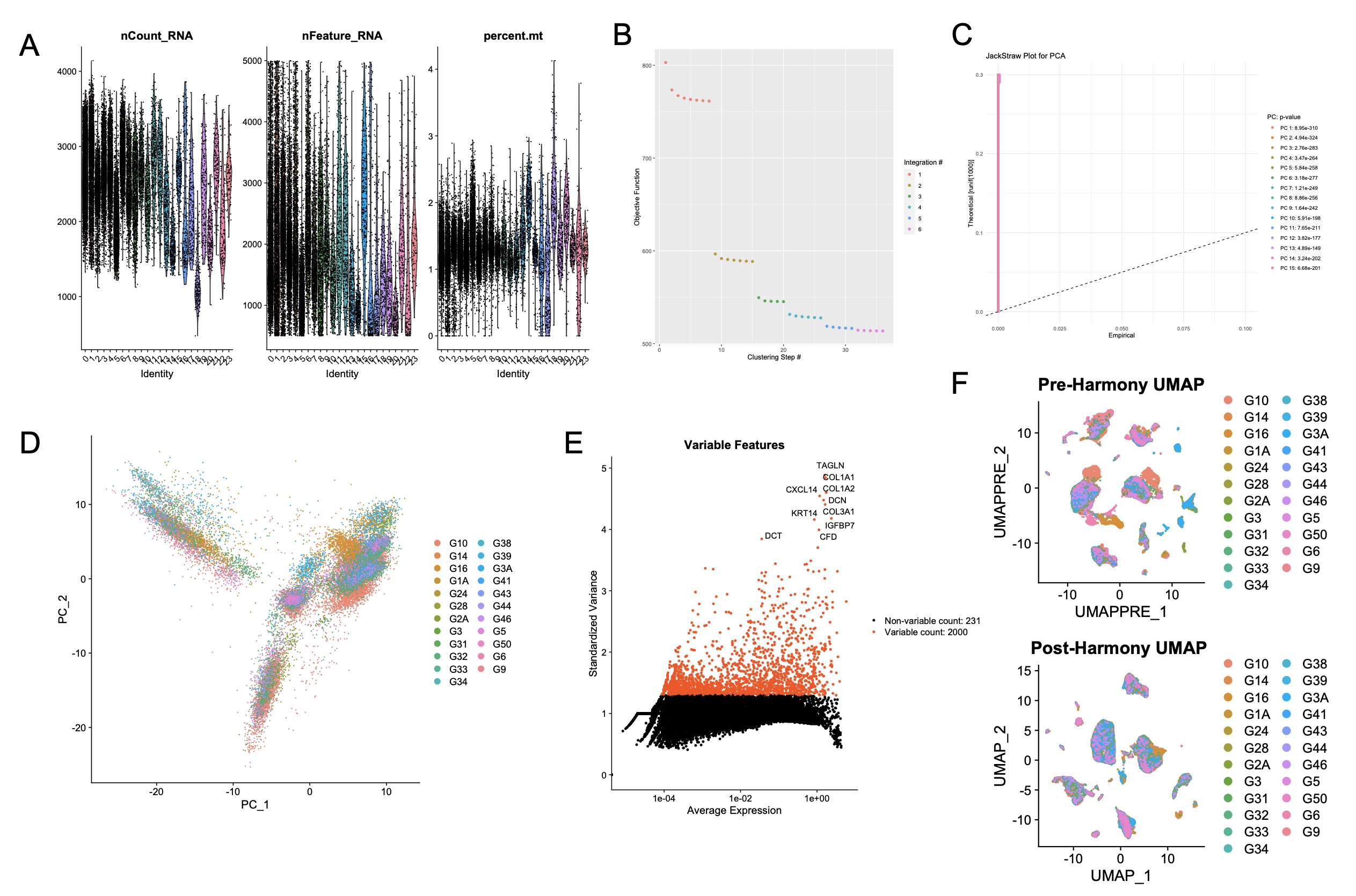

Supplement: Supplementary file 10 [file Image2.jpeg]

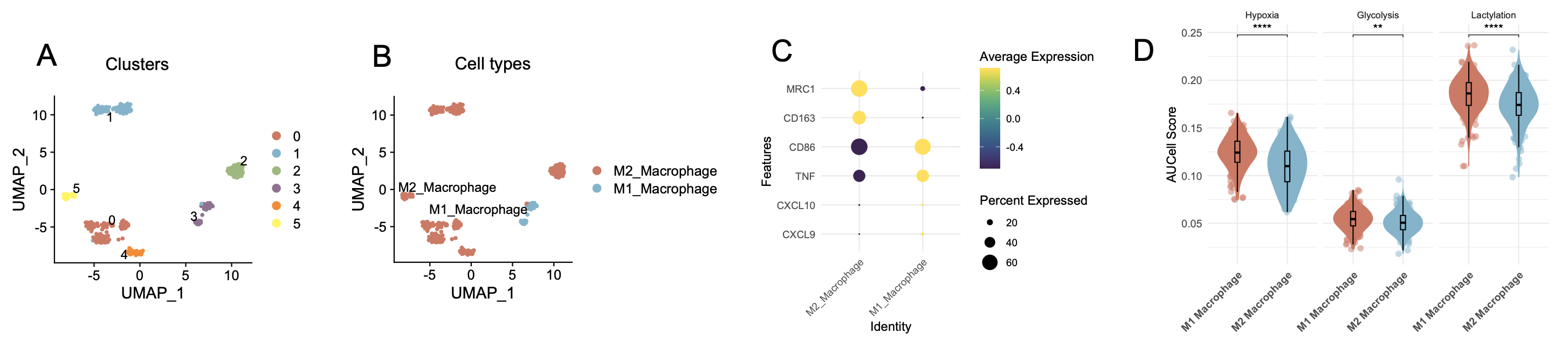

Supplement: Supplementary file 11 [file Image5.jpeg]

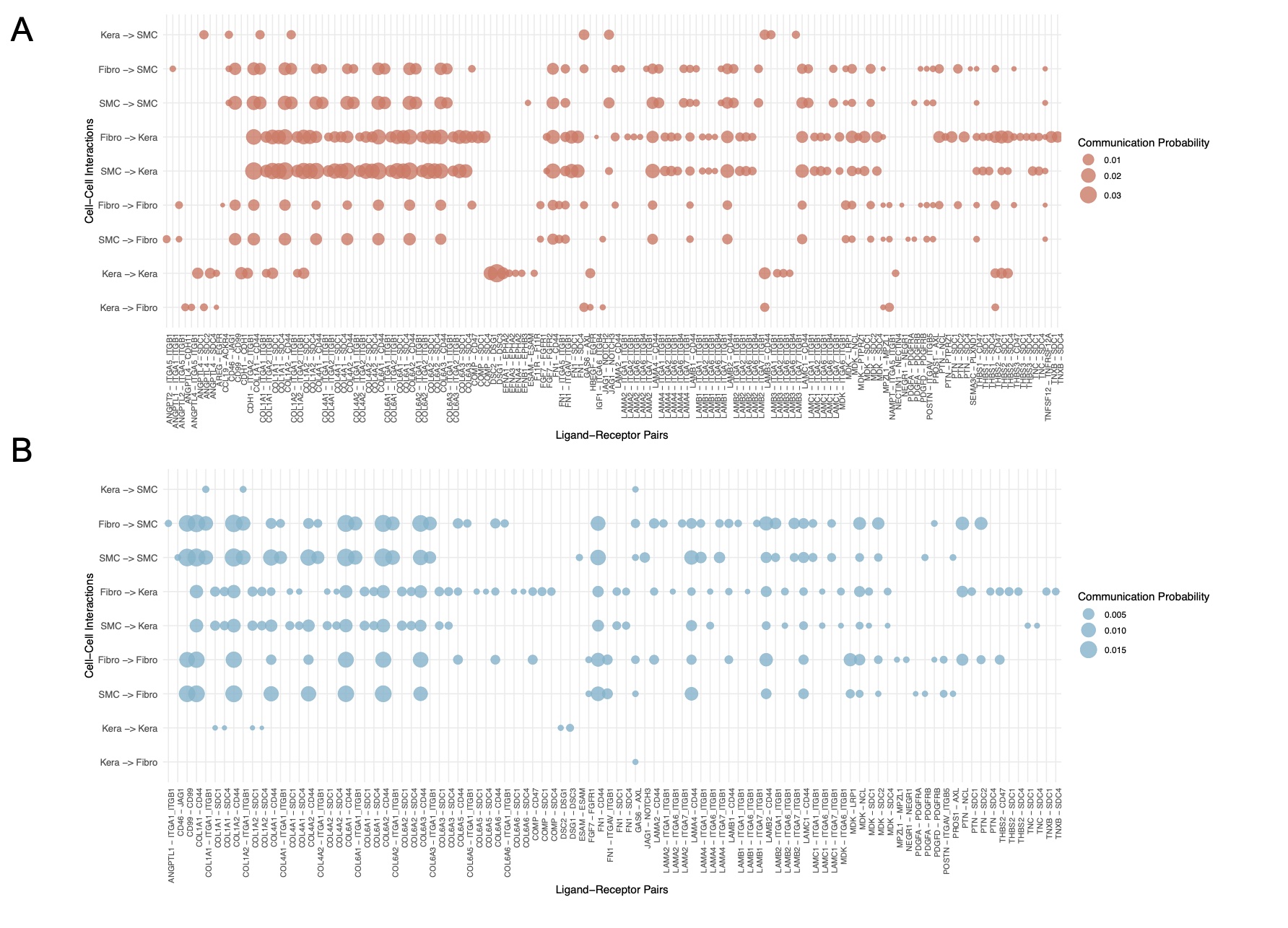

Supplement: Supplementary file 16 [file Image8.jpeg]

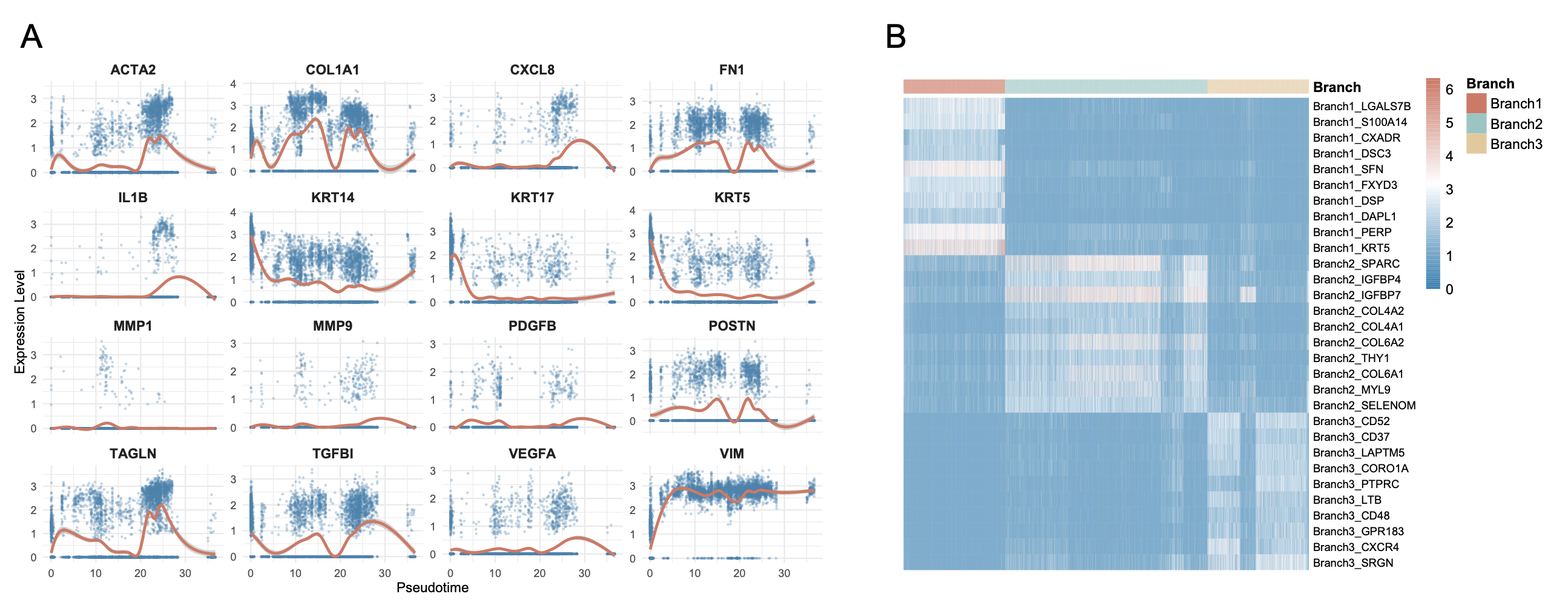

Supplement: Supplementary file 21 [file Image6.jpeg]
