# Supplementary material for: A comprehensive characterization of metabolic signatures—hypoxia, glycolysis, and lactylation—in non-healing diabetic foot ulcers
Source: Front Mol Biosci. 2025 Jul 9;12:1593390. doi: 10.3389/fmolb.2025.1593390 (PMC12284369; doi:10.3389/fmolb.2025.1593390)
Supplement: Supplementary file 4 [file Table1.docx]

# Supplementary Table 1. Cell Type Annotation and Marker Genes

| **Cell Type** | **Abbreviation** | **Representative Marker Genes** |
| --- | --- | --- |
| Smooth muscle cells | SMCs | TAGLN+, ACTA2+ |
| Fibroblasts | Fibro | DCN+, CFD+ |
| Vascular endothelial cells | VasEndo | ACKR1+ |
| Lymphatic endothelial cells | LymphEndo | CCL21+ |
| T lymphocytes | T-lympho | CD3D+ |
| Macrophages | Macro | IL1B+, CD163+, MRC+, TNF+, CD86+, CXCL9+, CXCL10+ |
| Keratinocytes | Kera | KRT1+, KRT10+, KRT5+, KRT14+ |
| Natural killer cells | NK | CCL5+, GZMB+ |
| NKT cells | NKT | CD3D+, CCL5+ |
| Melanocytes / Schwann cells | Melano/Schwann | MLANA+, CDH19+ |
| Sweat / Sebaceous gland cells | Sweat/Seba | DCD+ |
| Erythrocytes | Erythro | HBB+ |
| Dendritic / Langerhans cells | DCs | GZMB+, IRF8+ |
| B lymphocytes | B-lympho | CD79A+, MS4A1+ |
| Mast cells | Mast | TPSAB1+ |
| Plasma cells | Plasma | MZB1+ |
